# A scoping study of interventions to increase the uptake of physical activity (PA) amongst individuals with mild-to-moderate depression (MMD)

**DOI:** 10.1186/s12889-018-5270-7

**Published:** 2018-03-21

**Authors:** Katarzyna Karolina Machaczek, Peter Allmark, Elizabeth Goyder, Gordon Grant, Tom Ricketts, Nick Pollard, Andrew Booth, Deborah Harrop, Stephanie de-la Haye, Karen Collins, Geoff Green

**Affiliations:** 1 Collegiate Crescent, Sheffield Hallam University, S10 2BP, Sheffield, UK; 2 ScHARR, The University of Sheffield, Regent Court, 30 Regent Street, Sheffield, S1 4DA UK; 3 Survivors of Depression in Transition (SODIT), Jessel Street, Sheffield, S9 3HY UK

## Abstract

**Background:**

Depression is the largest contributor to disease burden globally. The evidence favouring physical activity as a treatment for mild-to-moderate depression is extensive and relatively uncontested. It is unclear, however, how to increase an uptake of physical activity amongst individuals experiencing mild-to-moderate depression. This leaves professionals with no guidance on how to help people experiencing mild-to-moderate depression to take up physical activity. The purpose of this study was to scope the evidence on interventions to increase the uptake of physical activity amongst individuals experiencing mild-to-moderate depression, and to develop a model of the mechanisms by which they are hypothesised to work.

**Methods:**

A scoping study was designed to include a review of primary studies, grey literature and six consultation exercises; two with individuals with experience of depression, two pre-project consultations with physical activity, mental health and literature review experts, one with public health experts, and one with community engagement experts.

**Results:**

Ten papers met the inclusion criteria and were included in the review. Consultation exercises provided insights into the mechanisms of an uptake of physical activity amongst individuals experiencing mild-to-moderate depression; evidence concerning those mechanisms is (a) fragmented in terms of design and purpose; (b) of varied quality; (c) rarely explicit about the mechanisms through which the interventions are thought to work. Physical, environmental and social factors that may represent mediating variables in the uptake of physical activity amongst people experiencing mild-to-moderate depression are largely absent from studies.

**Conclusions:**

An explanatory model was developed. This represents mild-to-moderate depression as interfering with (a) the motivation to take part in physical activity and (b) the volition that it is required to take part in physical activity. Therefore, both motivational and volitional elements are important in any intervention to increase physical activity in people with mild-to-moderate depression. Furthermore, mild-to-moderate depression-specific factors need to be tackled in any physical activity initiative, via psychological treatments such as Cognitive Behavioural Therapy. We argue that the social and environmental contexts of interventions also need attention.

## Background

Depression is the largest contributor to disease burden globally, with around 300 million people affected [1]. It is diagnosed by the presence of a range of symptoms that are not due to other conditions, including insomnia, fatigue, and loss of interest in activities which were once enjoyable (anhedonia) [2]. Depression can be episodic and vary in severity. The distinction between mild, moderate and severe depression is made largely on the number of symptoms; five or more usually termed severe or major depression, less than five, mild or moderate [3]. The episodic nature of the condition can make planning, anticipating and sustaining activities difficult for people. This is likely to be a major factor in shaping adherence as well as an uptake of physical activity (PA).

PA encompasses everyday activities (such as cycling or walking), work-related activities, housework, do-it-yourself or gardening, and recreational activities such as dancing, active games, organised sport and gym work [4].

The UK’s NICE guideline for depression [2] highlights the cost-effectiveness of a structured group exercise programme as an adjunct treatment for mild-to-moderate depression (MMD); it recommends that individuals with MMD engage in three sessions of 45–60 min of PA per week, over 10 to 14 weeks. A report for the National Service Framework for Mental Health also recommends PA as a treatment option for people with depression [5]; however, neither offers detailed guidelines for the implementation of the recommendation and there is little consensus in the literature.

The overarching goal of this scoping study was to systematically map the literature on the topic i.e. interventions to increase the uptake of physical activity amongst individuals with mild-to-moderate depression; identify key concepts; theories; sources of evidence and gaps in knowledge [6]. The study had five objectives: (1) to identify interventions which sought to increase the uptake of PA in people with MMD; (2) to identify the characteristics of these interventions, including modifications made for MMD; (3) to describe theories underpinning these modifications; (4) to identify barriers and enablers to the uptake of PA in people with MMD; and (5) to develop an initial conceptual framework in the form of a model setting out the mechanisms by which interventions can be hypothesised to work, drawing on findings from literature and consultation exercises with the key stakeholders.

## Methods

Scoping study methodology [6, 7] was appropriate here as the study addressed an exploratory question in the public health field involving complex multi-factorial interventions with a scarcity of high-quality randomized controlled trial (RCT) evidence [7].

The study was undertaken between May 2016 and January 2017 and was based on the framework by Levac and colleagues [8] that systematises a process of undertaking a scoping study into six stages; these are used as headings below, we added a seventh stage, the development of a model.

### Stage 1: Identifying the research question

Consultation was an ongoing process throughout the study [9]. Key stakeholders were approached at the outset and contributed to the establishment of the research question and overall purpose of the study (further information about the consultation can be found in the consultation section). The research question established in this way was:

What are the characteristics of the interventions that aim to increase the uptake of physical activity amongst individuals with mild-to-moderate depression?

To address this question the following objectives were developed:

- To gather data concerning interventions developed to increase an uptake of PA amongst individuals with MMD, with specific focus on the MMD-related modifications, the theories on which these modifications are based, and barriers and enablers to the uptake of PA amongst people experiencing MMD.
- To develop a model of how approaches to increase the uptake of PA amongst people with MMD can be hypothesised to work.

### Stage 2 study eligibility for inclusion in the review

The PICOS framework was used to develop the search question and clarify exclusion/inclusion criteria [10]. The framework supports the construction of an effective combination of search terms through the categorisation of search terms into the concepts of Population, Intervention, Comparison, Outcomes and Study Design [11, 12]; such an approach also helps to ensure that the searches are comprehensive and reduces the risk of bias.

***Population:*** Adults with MMD (main group or subgroup).

***Intervention:*** Interventions developed to increase the uptake of PA in individuals with MMD (either as a main group or subgroup).

***Comparator:*** People with MMD receiving treatment as usual or, as controls, individuals with no depression.

***Outcome:*** Uptake of physical activity behaviour.

**Study type:** Studies reporting primary data and published in the English Language.

The positive effects of PA on alleviating depression symptoms were taken as uncontested [13–16], hence studies exploring this were excluded.

### Stage 3 identifying studies relevant to the research question

As a scoping study, two specific limitations were put on the search. The first was the decision to search only three databases, MEDLINE, PubMed, and PsycINFO (ProQuest). These databases were selected as their scope best fitted the remit of the review. The second was to search for papers published between January 2001 and January 2017. The start date of 2001 was selected in line with the publication of the National Quality Assurance Framework for exercise referral, intended to raise standards of exercise referral schemes, and consequently to increase physical activity levels in the population [17]. The search terms included: access/accessibility, active play, depression, depressive disorder, physical fitness, physical activity, exercise, exercise therapy, referral, self-referral, referred, health behaviour, health promotion, public health, physical environment, and social environment. These terms were developed by an experienced Information Scientist (DH), who also identified key and appropriate databases. She designed and ran the initial search strategy, from a small number of relevant articles identified in the consultation process. The initial search strategy was then reviewed and refined by other members of the team (KM and PA). The searches were re-run before the final analysis commenced.

The search for grey literature was informed by Frank’s et al. [18] process. Searches were performed on trial databases (e.g. www.isrctn.com, grey literature databases (e.g. www.opengrey.eu), websites of relevant key organisations, and an Internet search engine (Google Scholar). Citation searches were also undertaken.

Website searches of key organisations were conducted, including the National Institute for Health and Care Excellence (NICE), the UK’s Department of Health (DoH), the World Health Organisation (WHO), the King’s Fund, MIND, Mental Health Research UK, and the Mental Health Foundation. In addition, a number of organisations were contacted, including Local Authorities, the local ‘Improving Access to Psychological Therapies’ (IAPT) services; and the National Centre for Sport and Exercise Medicine (NCSEM).

### Stage 4: Charting of information and data within the included studies

In line with the scoping review methodology, a formal quality assessment of the studies was not required [19]. Two data extraction tables were created that included: Table 1: details of the studies and participants: author and year, country, study type, setting, conditions, diagnosis methods, number of participants, age and sex; Table 2: details of the interventions: author and year, types of PA, intensity, duration of intervention, whether or not and, if so, how an intervention was modified for individuals with depression, motivational component, how PA was assessed, delivery mode and outcomes. We also extracted information about the theory on which the intervention was based (Table 3); and barriers and enablers to an uptake of PA amongst individuals with MMD.

**Table 1:** Study; Country Study type; Setting; Conditions; Diagnosis tool; Number of participants; Age; and Sex

Author	Country	Study type	Setting	Conditions (%) Provided, whenever available, if an interventions was delivered to a mixed clinical group	Diagnosis tool	Number of participants	Age	Sex
Forsyth et al., 2009 [64]	Australia	Pilot (feasibility) RCT	Primary Care	a) Depression (51%) b) Anxiety (19%) c) Mixed anxiety-depression disorder (30%) Participants’ mean BMI = 29.7 kg/m2	The Depression Anxiety Stress Scale DASS-21	At baseline = 25; but only 18 patients completed an initial assessment At week 12 = 5	Age range: 19–73	At baseline: Male = 9 Female = 16
Mailey et al., 2010 [25]	USA	Pilot RCT	Community and University-based Healthcare Services	a) Depression b) Anxiety	The Beck’s Depression Inventory (BDI)	Intervention arm At baseline = 26 At week 10 = 23 Control arm At baseline = 25 At week 10 = 24	At baseline: Intervention & Control Arms = 25 yr (18–52 yr)	At baseline: The sample (in both study arms) was primary female (68.1%)
Oeland et al., 2010 [31]	Denmark	RCT	Primary Care	a) Mild-to-moderate depression (MMD) b) MMD recurrent c) Anxiety	The Hamilton Depression Rating Scale (HAM-D)	Intervention Arm At baseline: Total number of Pts = 27 [*MMD* (60%); *MMD recurrent* (18%); *Anxiety* (23%)] At week 32: Total number of Pts = 13 [condition-specific information - not provided] Control Arm Total number of Pts: 21 [*MMD* (43%); *MMD recurrent* (38%); *Anxiety* (20%)] At week 32: Total number of Pts = 15 [condition-specific information - not provided]	At baseline: Intervention arm = 36 yr (18–52) Control arm = 40 yr (20–67)	At baseline: Intervention arm = 85/15 Control arm = 67/33
Pentecost et al., 2015 [32]	UK	Pilot RCT	Primary Care: Improving Access to Psychological Therapies (IAPT) Services	a) Mild Depression b) Moderate Depression c) Severe Depression	The Clinical Interview Schedule - Revised (CIS-R) & the Patient Health Questionnaire-9 (PHQ-9)	Intervention 1 arm At baseline: (Behavioural activation plus physical activity promotion) *Mild depression* = 6 (20%) *Moderate depression* = 16 (53.3%) *Severe depression* = 8 (26.7%) At week 16: *Mild depression* = 4 (13.3) *Moderate depression* = 16 (53.3%) *Severe depression* = 10 (33.3%) Intervention 2 arm (Behavioural activation) At baseline: *Mild depression* = 2 (9.1%) *Moderate depression* = 2 (9.1%) *Severe depression* = 1 (4.5%) At week 16: *Mild depression* = 1 (4.8%) *Moderate depression* = 4 (19%) *Severe depression* = 3 (14.3%)	At baseline: Intervention 1 arm 18–30 yr, *n* = 6 31+ yr, *n* = 24 Intervention 2 arm 18–30 yr, n (%) = 6 31+ yr, n = 24	At baseline: Intervention 1 arm Male = 18 Female = 20 Intervention 2 arm Male = 13 Female = 17
Piette et al., 2011 [26]	USA	RCT	Various, a community-university-and VA healthcare system	Comorbid moderate depression (Beck Depression Inventory scores ≥14) & diabetes	The Beck’s Depression Inventory (BDI)	Intervention arm At baseline = 172 At 12 months = 145 Control arm At baseline = 167 At 12 months = 146	At baseline: Patients’ mean age was 56 yr	At baseline: Male = 49% Female = 51%
Suija et al., 2009 [27]	Estonia	RCT	Primary Care	Mild-to-moderate depression (MMD)	The Composite International Diagnostic Interview (CIDI)	Intervention arm (patients with depression) At baseline = 48 randomised; 16 agreed to participate in the study At week 24 = 4 Control arm (non-depressed patients) At baseline = 58 randomised; 5 agreed to participate in the study At week 24 = 5	At baseline: 18–29 yr, *n* = 7 40–59 yr, *n* = 5 ≥60 yr, n = 1	At baseline: Male = 1 Female = 15
Crone et al., 2008 [33]	UK	Quasi-experimental	Primary Care	Mental health group (4.6% of all study participants); this included: a) Depression (61%) b) Anxiety/loss of confidence (26%) c) Stress/tension (13%) Physical health: Cardio-vascular disease, overweight, obesity, diabetes, musculoskeletal health, unfit/sedentary, or other	No information given	At baseline: Mental health = 134 Physical health = 2500 At 12 week or programme completion: Mental health = 29 Physical health = 935	At baseline: Mental health group: 42 ± 14 yr Physical health group: 51 ± 14 yr	At baseline: Male = 36% Female = 64%
Duda et al., 2014 [28]	UK	RCT	Primary Care	Mental health group: a) Probable (Mild) Depression (18.9%) b) Probable anxiety (34.8%) Comorbidities: Two or more factors for coronary heart disease (CHD), overweight, obesity, other long term conditions (LTCs), asthma, bronchitis, diabetes, those for whom regular PA may prevent the onset of osteoporosis, those with borderline hypertension.	The Hospital Anxiety and Depression Scale (HADS)	Total No of participants: 347 (a sample of 494 participants was required to detect a difference in mean PA time across the intervention and control arms) Intervention arm At baseline = 184 At 6-month follow up = 82 Control arm At baseline = 163 At week = 92	At baseline: Intervention arm < 30 yr, *n* = 19 30–49 yr, *n* = 76 50–64 yr, *n* = 64 ≥65 yr, *n* = 25 Control arm < 30 yr, *n* = 11 30–49 yr, *n* = 77 50–64 yr, *n* = 50 ≥65 yr, n = 25	At baseline: Intervention arm Male = 45 (24.5%) Female = 139 (75.5%) Control arm Male = 49 (30.1%) Female = 114 (69.9%)
Littlecott et al., 2014 [29]	UK	RCT	Primary Care	Mental health (4%): a) Depression b) Anxiety Physical health: a) CHD risk factors Both: Comorbid mental health and physical health	The Hospital Anxiety and Depression Scale (HADS)	At baseline, 1080 participants were randomised to each trial arm Intervention arm At 12 months: Mental health =19 CHD risk = 362 Control arm At 12 months: Mental health = 13 CHD risk = 339	At baseline: 16–44 yr, *n* = 191 45–59 yr, *n* = 303 ≥60 yr, n 386 Condition-specific age data is not provided	At baseline: Male = 316 Female = 590 Condition-specific sex data is not provided
Pomp et al., 2013 [30]	Germany	Quasi-experimental	Orthopaedic rehabilitation	Depression (10%) Other health conditions - no information available	The Patient Health Questionnaire-9 (PHQ-9)	Intervention arm At baseline = 227 At week 6 = 132 Control arm At baseline = 279 At week 6 = 229	The authors state that the control and intervention arms did not differ in terms of sex and age. No further details are provided.	The authors state that the control and intervention arms did not differ in terms of sex and age. No further details are provided.

**Table 2:** Study; Types of PA; Intensity of PA; Duration of intervention; Modified for depression?; Motivational component?; PA assessment; Delivery mode; and Outcome

Study	Types of PA	Intensity of PA	Duration of intervention	Modified for depression?	Motivational component	PA assessed and assessment method	Delivery mode	Outcome (re increasing an uptake of PA amongst those with depression)
Forsyth et al., 2009 [64]	Various e.g. waking; Some participants were referred to leisure facilities.	Information unavailable	12 weeks	Yes	Yes: Motivational Interviewing (MI)	Yes An indirect measure: muscular endurance and aerobic fitness tests	MC: Face-to-face PAC: Mainly unsupervised	Successful: The intervention was successful in increasing the participants’ muscular endurance and aerobic fitness.
Mailey et al., 2010 [25]	Various e.g. walking	The participants were asked to fill in an activity log to report on the perceived intensity of PA	10 weeks	Yes	Yes: Social Cognitive Theory (SCT) Four modules with components addressing barriers to the uptake and maintenance of PA. *Module 1.* Getting Started: covered the benefits of exercise; *Module 2.* Planning for Success: introduced self-efficacy, outcome expectations and goal setting; *Module 3.* Beating the Odds: looked at barriers to PA and the ways of overcoming them; *Module* 4. Sticking with it: provided guidance on maintenance.	Yes An objective assessment: Pedometer Plus a subjective, self-reported, measure: An activity log for monitoring: a) PA type Perceived exertion during PA	MC: Internet-based plus two monthly meetings with PA counsellors PAC: Unsupervised	Successful: The intervention showed statistically significant improvement in both, the control and intervention conditions. However, the exercise self-efficacy declined over the duration of the intervention, but more so in the control than intervention condition.
Oeland et al., 2010 [31]	Supervised sessions: 1) Aerobic training of cardiorespiratory functioning 2) Weight lifting: 5 basic exercises for leg, chest, abdomen, and lower and upper back muscles. Home-based physical activity	1) High intensity aerobic exercises: 65%–75% of maximum aerobic capacity 2) Intensity: 10 RM (repetition max) 3) Home-based physical activity - intensity not provided Supervised sessions: 2 x week Home-based PA: 1 x week	20 weeks	Yes	No	Yes A subjective, self-reported, measure of PA: The International Physical Activity Questionnaire short version Plus an indirect measure of PA: VO2	MC: N/A PAC: Face-to-face A structured and supervised group PA programme Plus one unsupervised PA session per week	Successful but low uptake: The interventions showed significant improvements in levels of PA as measured by VO2 but the uptake of the intervention was low.
Pentecost et al., 2015 [32]	Various, e.g. walking, gardening, dancing, swimming, gym-based PA	Intensity of aerobic exercise & strength training was measured Plus a subjective measure: self-reported intensity of PA 1) Sedentary, 2) light PA 3) Moderate PA, 4) vigorous, 5) moderate and vigorous	16–20 weeks	Yes	Yes The participants were randomly allocated to Behavioural Activation (BA) or Behavioural Activation plus physical activity promotion (BAcPAc) intervention arm.	Yes An objective assessment: Pedometer Plus a subjective, measure: self-reported intensity of PA: ‘light’, ‘moderate’ or ‘vigorous’, recorded in a diary	MC: Face-to-face, over the telephone or the combination of both An initial assessment, plus up to 12 support sessions with PWPs. Plus a written self-help booklet based on BA protocol. PAC: Unsupervised	Unsuccessful: The engagement of IAPT practitioners and hence, participant recruitment, proved challenging.
Piette et al., 2011 [26]	Walking	Information unavailable	12 months in total: 12 weeks weekly sessions plus nine monthly booster sessions	Yes	Yes: Cognitive Behavioural Therapy (CBT)	Yes An objective assessment: Pedometer	MC: Over the telephone or face-to-face PAC: Unsupervised	Successful: The intervention was successful in increasing an uptake of PA.
Suija et al., 2009 [27]	Nordic Walking	Information unavailable	24 weeks	Yes	No	Yes A subjective, self-reported, measure: PA diaries Plus the physical fitness assessment: 2 km walking test	MC: N/A PAC: Unsupervised	Unsuccessful: No improvements in an uptake and levels of PA; only 4 depressed participants completed the intervention.
Crone et al., 2008 [33]	Gym-based PA	Information unavailable	8–12 weeks	No	No	Yes The researchers monitored the number of PA sessions attended by the participants: Attenders (< 80% attendance) Completers (≥80% attendance)	Pre-entering the PA programme: Face-to-face referral by a healthcare professional (general practitioners, GP; practice nurse; physiotherapist; or other: dietitians, psychiatrists, nurse specialists, cardiac nurses, health visitors, smoking cessation officers, healthy lifestyle coordinators), to a local leisure centre MC: N/A PAC: One-to-one consultations with an exercise professional	Unsuccessful: Embedded within PARS; the study compared outcomes of uptake, attendance and completion of the programme between patients in two groups (Group 1: Mental Health; Group 2: Physical Health). Referrals with a mental health condition had poorer attendance and completion rates that those referred with a physical health condition.
Duda et al., 2014 [28]	Outdoors (e.g. walking) plus Gym-based PA	Time spent in moderate or vigorous PA was recorded,	8–12 weeks	No	Yes: Self-Determination Theory (SDT) It compared two types of PARS, a standard provision and the SDT-based.	Yes A subjective, self-reported, measure: The 7-Day Physical Activity Recall	Pre-entering the PA programme: Face-to-face Individuals enrolled by their GPs or practice nurse to an exercise referral scheme. MC: The initial consultation with SDT-trained health and fitness advisors (HTA): Face-to-face An additional 2 brief interactions with HTA: Face-to-face or over the telephone The final consultation with HTA: Face-to-face PAC: One-to-one consultation with an exercise professional	Unsuccessful in the sense that there was no difference in activity levels between the two arms of the study; as such the intervention made no difference over standard provision. However, it is worth noting that physical activity increased and depression improved in both arms.
Littlecott et al., 2014 [29]	Gym-based PA	The perceived intensity of PA was assessed (moderate intensity or greater intensity, where ‘moderate’ was defined as how participants feel when walking at a normal pace)	6–19 weeks (intended duration 16 weeks)	No	Yes: the integrated Self-Determination Theory (SDT), Self-Efficacy Theory (SET), and social support	Yes A subjective, self-reported, measure: The General Practice Physical Activity Questionnaire (GPPAQ)	Pre-entering the PA programme: Face-to-face referral by healthcare professional MC: The PARS MC component (based on SDT and SET):Information unavailable; reported elsewhere Support from family and friends. PAC: One-to-one consultation with an exercise professional; Supervised group-based activity	Unsuccessful: There was some statistically significant improvement in levels of PA post-intervention but only in the coronary heart disease (CHD) group. Adherence was poor amongst mental health patients.
Pomp et al., 2013 [30]	Various e.g. swimming, running,	Self-reported; the perceived intensity of PA (i.e. moderate or strenuous)	6 weeks	No	Yes: Self-Regulation The intervention included an encouragement to form 5 post-rehabilitation action plans (where and when), and to generate post-rehabilitation physical activity ideas (types of PA). In addition, the intervention included the volitional strategy of action control.	Yes A subjective, self-reported, measure: A modified version of the Godin Leisure-Time Exercise Questionnaire (GLTEQ), plus a PA diary	MC: Computer-based PAC: Unsupervised	Unsuccessful: A computer-based self-regulation intervention to increase PA/engage in regular PA after discharge from the orthopaedic clinics, and the researchers were interested in whether or not depression limits the usefulness of this programme. Without modification for depression, the intervention did not work.

**Table 3:** Conceptual frameworks of interventions which included a psychological component

Approach/study	Approach or Theory/theories on which the modification has been based	Conceptual mechanisms of change	Details of intervention and depression specific elements (if any).
Motivational Interviewing (MI) [21]	The study employed Motivational Interviewing (MI) [37], and it used a goal-based approach in identifying patient readiness to change for diet and physical activity behaviours [38].	MI is a “client centred, directive method for enhancing intrinsic motivation to change by exploring and resolving ambivalence” [39] p. 25. MI comprises of two main components: (a) increasing an individual’s motivation to change behaviour; (b) increasing an individual’s commitment to change. MI draws explicitly and implicitly on a number of behaviour change conceptual frameworks [40]. Goal setting is based on self-regulation theory and control theory. Goal theory, focuses on mechanisms, which make it possible for intention to be translated into action. The mechanisms to enhance one’s ability to perform behaviour are, amongst others, self-monitoring or setting realistic goals [40].	Consultations s with exercise professionals were underpinned by a motivational interviewing (MI) approach and included goal setting. The short-term goals developed by participants included homework activities, which were reviewed at the beginning of the subsequent consultation [41]. The use of homework, including scheduling daily activities (‘therapeutic homework administration procedure’), was a depression-specific modification of the intervention. The use of homework has been recognised as effective in the treatment of mental illness [41] and planning daily activity can be as effective as Cognitive Behavioural Therapy (CBT) and other psychological treatments in alleviating depression symptoms. Treatment fidelity revealed, however, that these components of the interventions were not fully delivered.
Intervention based on the principles of SCT [23]	Social-Cognitive Theory (SCT)	SCT assumes that self-efficacy (confidence to perform a particular behaviour; perceptions about one’s own capabilities) is the key determinant of behaviour [42]. Self-efficacy expectations are beliefs about one’s ability to perform behaviour irrespective of the external circumstances [42]. Social influences and expectation of the outcomes of behaviour are other determinants of whether or not one will attempt to change [42]. According to SCT self-efficacy can be enhanced by: (i) mastery experience - taking small steps which lead to mastering a skill; (ii) vicarious learning – learning occurs through observing others; (iii) verbal persuasion and believing that one’s have what is required to succeed; (iv) affective states – dealing with negative emotions through various techniques [42].	It was a 10-week internet-based physical activity intervention and it included 4 modules with components addressing barriers to the initiation and maintenance of physical activity. Specifically, Module 1 *Getting Started* included information about the benefits of exercise; Module 2 *Planning for Success* introduced self-efficacy, outcome expectations and goal setting; Module 3 *Beating the Odds* looked at barriers to physical activity and looked at the ways of overcoming them; Module 4 *Sticking with It* provided guidance on maintenance.
Behavioural activation (BA) [20]	Behavioural Activation (BA) [43] is *grounded* in learning theory and contextual functionalism. The study used two modifications: behavioural activation (BA) and behavioural activation plus physical activity promotion (BAcPAc).	BA [43] is a development of activity scheduling, which is a CBT component. Two mechanisms of affecting change: 1. Using avoided activities as a guide for activity scheduling (PA can be one of those activities). That is, scheduling daily activities consistently with avoided activities but consistent with one’s *valued direction.* 2. Functional analysis of cognitive processes, which lead to activity avoidance. The therapy focuses on the entire event and factors that may affect the occurrence of negative responses. Contextualisation explores what factors predict and maintain negative responses [44]. A developmental formulation is established which explores how social context has affected a depressed individuals copying behaviour. Alternative approaches to creating one’s responses is developed [44].	BA activation has been proposed as a treatment for depression and as the basis for interventions to increase physical activity levels.
Intervention based on the CBT principles [19]	Cognitive Behavioural Therapy (CBT) CBT combines Cognitive Therapy (CT) [46] and Behaviour Therapy (BT) [48]. The CBT programme comprised 12-weekly sessions followed by 9 monthly booster sessions.	One could tackle a health-related behaviour by examining processes (hidden motivation and otherwise), which lie at the root of the problem. Changing self-referent negative thinking, which promotes low mood, may improve motivational and behavioural features. CBT enables individuals to develop better coping skills for dealing with negative self-referent thought, believes and attitudes, which, in turn, affect their feelings and behaviours (e.g. including PA). It comprises activity scheduling and cognitive challenges to negative thoughts, core beliefs and assumptions [44].	At the outset, the aim of the CBT sessions was to address patients’ depressive symptoms; after five sessions, the nurses delivering the interventions initiated discussions about a walking programmes and links between depression and PA. A manual was used to provide step-by-step visual instructions to facilitate sessions; it included elements common in depression CBT manuals plus additional concepts related to diabetes self-care and PA.
Intervention based on the principles of SDT [15, 16] Intervention based on the principles of SDT plus an MI element [27]	Self-Determination Theory (SDT) Exercise Referral Schemes are based on multiple theories. The studies included in this review explored such concepts as Self-Efficacy and Self-Determination Theories, and their effects on PA behaviour.	SDT focuses on both, the determinants and consequences of autonomous (e.g. personal values) and controls motives; it may promote more autonomous motivation, which has been found important in interventions for individuals with depression. It highlights the importance of feeling competent, in control and connected with others [27]. It assumes that high levels of autonomous motivation are link to finding PA intrinsically enjoyable or, at least, connected to desired outcomes [27].	Interventions based on SDT were not modified for individuals with depression. The researchers found that the intervention was effective in increasing physical activity levels in the cardiac group but not in the depression group. This suggests that unmodified interventions may be ineffective or less effective in depressed patients.
Intervention based on the Energy and Strength Model [24]	The study used the Strength and Energy Model [49, 50]; implementation intention and planning, self-efficacy and action control [51, 52].	The strength and energy model assumes that self-regulation is a global energy that is utilised on self-regulated activities in different areas of action. As a self-regulation is represented as a limited source, self-regulation in one area may lead to ego depletion, and a failure to self-regulate in the other areas. The regulation of depression symptoms may lead to reduction of self-regulation energy and difficulties in using self-regulation in the other areas, such as physically activity.	The intervention itself was designed for orthopaedic patients. The researchers were interested in whether depression limits usefulness of this programme. They concluded that depression did modify the effectiveness of the programme. They concluded: “a self-regulation intervention, which is not tailored to the needs of the individuals suffering from depressive symptoms, might not be effective…” [24] p. 7.

### Stage 5: Collating, summarising and reporting results of the review

An analytical descriptive method was used to chart the data and to extract contextual or process-oriented information from each study [8]. This stage also included a qualitative data analysis approach [20]. The qualitative analysis focused mainly on modifications of interventions made for people experiencing MMD, the theories on which these modifications were based, and the barriers and enablers. This stage included consideration of implications for future research, policy and practice.

### Stage 6: Consultation

There were six consultation exercises: two meetings with the lay representatives/individuals with experience of depression; two pre-project consultations with physical activity and mental health and information specialists; one pre-project consultation with public health specialists; and one consultation meeting with community engagement experts. Twenty stakeholders participated in the study, including individuals with experience of depression (*n* = 6), mental health (*n* = 2) and public health practitioners (n = 2), and academic experts in the fields as follows: physical activity (n = 2), public health (*n* = 3), mental health (n = 2), literature review (*n* = 1), and community engagement (n = 2). Efforts were made to ensure relevant and multi-disciplinary representation of experts to cover the various aspects of such a multi-disciplinary intervention which an uptake of PA amongst individuals with depression represents.

Meetings included a combination of structured presentation from the research team of the key issues that were drawn out from the literature review and group discussions. The discussions were digitally recorded and were transcribed verbatim. The purpose of the consultation exercises was twofold: 1) to integrate stakeholders into the entire research process, including deciding on the scope of the study, interpreting the findings from the literature review, developing a model of an uptake of PA amongst individuals with MMD, and knowledge translation, and, 2) to consider the implications of the findings for future practice and research, including intervention development.

### Data analysis

A thematic analysis of the data from consultation transcripts was undertaken [21]. Thematic analysis was chosen as it supports flexibility in the analysis of research data in a couple of ways, i.e. inductive and deductive [22, 23], while allowing the researchers to provide a thorough account of the data. The data were independently coded by two researchers (KM and PA). The analysis of data began with an initial framework inductively developed using the literature review-elicited themes and categories regarding the key factors, which may affect the uptake of PA amongst individuals experiencing MMD. The initial framework was then refined further through iteration as coding progressed. The inter-coder agreement ranged from 83% to 91%, with a mean score of 87%; any discrepancies in judgement were resolved through discussion. The final themes were discussed and agreed upon by the entire research team.

## Results

### Literature review

**Fig. 1:**
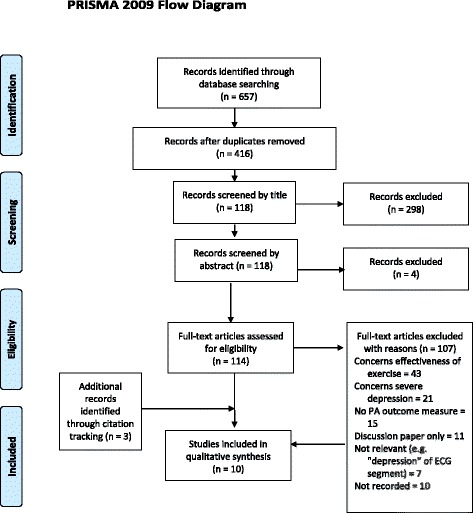
Prisma flow diagram

A PRISMA flowchart summarising the search and screening process of databases, including primary studies, trials and grey literature searches, is shown in Fig. 1.

The database searches returned 416 papers (after the removal of duplicates) that were reviewed by title and abstract and which resulted in the retention of 114 papers. A full-text screening of the remaining papers resulted in the identification of 7 papers that met the eligibility criteria of the review [24–30]. Citation tracking using the included papers generated a further 3 papers [31–33], giving a total of 10. Papers which met the criteria for inclusion in the review are listed in Table 1 and Table 2.

1. Interventions that aim to increase the uptake of PA in people with MMD:

The interventions had been undertaken in a range of countries. Three papers reporting research from UK primary care were part of a larger assessment of the UK’s Physical Activity Referral Schemes (PARSs) [28, 29, 33].

Interventions targeted patients treated for depression [26–30, 32, 33], depression and anxiety disorders [24, 25]. Various instruments for screening, diagnosing and measuring the severity of depression were used in the studies.

The participants were predominantly middle-aged (45–65 years); however, one study recruited college-aged participants [25]. Across studies, there were differences in the samples involved; seven studies recruited primary care patients [24, 27–29, 31–33], one study involved individuals enrolled in a community, university, and VA healthcare system [26], one study recruited orthopaedic patients [30], and one colleague students [25].

Studies used a range of PA outcome measures. In four studies the outcome was self-reported PA [28–30, 33]. In six studies the reported outcome was objectively measured levels of PA such as pedometers [22–25]. In one study the authors measured changes in physical fitness and in muscle endurance [24].

The most common study type was an RCT [26–28, 31], or pilot RCT [24, 25, 32]; two papers reported quasi-experimental designs [30, 33].

The first six papers listed in the Tables 1 and 2 were delivered specifically to individuals with MMD or depression and anxiety [24–27, 31, 32]; the remaining four were delivered to a mixed group of which the proportion with MMD was small, between 4% [29] and 18.9% [28].

(2) The characteristics of the interventions, including modifications made for individuals experiencing MMD:

Four of the interventions included an element that was specifically focused on depression or, more precisely, an element in which the aim was to overcome the motivational barriers created by depression; all four of these studies were in the depression specific group [24, 25, 31, 32]. Each of these studies was based on a different theoretical framework, these being one, or a combination of, a Motivational Interviewing (MI) approach [24], Self-Determination Theory (SDT) plus an MI element [32], Cognitive Behavioural Therapy (CBT) [26], Social Cognitive Theory (SCT) [25], Behavioural Activation (BA) [32] and the strength-energy model of self-control combined with Implementation Intention [30].

Only two of the depression-specific studies which included a motivational element [25, 26] measured its effect on mediating variables affecting PA behaviour change, such as self-efficacy. The remaining studies measured the effect of the intervention on PA behaviour only. The CBT-based intervention reported significant increases in the participants’ self-efficacy for increasing their PA levels at follow up (p<.0001), compared to the control group [26]. The other, SCT-based intervention, reported increases in perceived self-efficacy during the intervention, which, however, declined over a 10-week period [25].

In three of the four studies which included a depression specific motivational element, the PA component was unsupervised; in one, however, physical activities were taken under the supervision of an exercise practitioner [32]. Two of the depression specific group did not have such an element but rather delivered a generic intervention, designed for the general population, to a group with depression. In Suija’s et al. [27] study, the depressed individuals were offered a Nordic walking intervention. In Oeland and colleagues’ study [31], the participants were offered a structured and supervised physical group exercise programme. None of the four interventions that were not depression-specific had any depression-related motivational element even though people experiencing depression were a subgroup in the studies.

Three of the four non-depression specific studies were reporting the UK’s Physical Activity Referral Schemes (PARSs) [28, 29, 33]. The PARS studies included in the review incorporated a motivational component in their interventions, albeit not depression-specific. One of those studies [29] explored mediating variables, including: perceptions of autonomy support, the degree to which an individual feels competent, relatedness, and autonomy needs satisfaction, intention to be active, and motivational regulations for PA.

Various types of physical activity and exercise were used in the studies. None of the studies discussed the effect of intensity on uptake, although in at least one case it might be argued that the intervention’s intensity could affect it [31].

Out of the six interventions delivered specifically for individuals with depression, four were successful in increasing uptake of PA [24–26, 31] whereas two were unsuccessful [27, 32]. The four unmodified interventions were not successful.

(3) Theories on which these modifications have been made:

Table 3 delineates the theories, which reflect modifications for individuals with MMD. Researchers rarely offered theoretical explanations for the mechanisms through which the interventions were hypothesised to work although in some cases it could be discerned.

(4) Barriers and enablers to the uptake of PA in people with MMD:

The review revealed a number of barriers to the uptake of PA. There was evidence that interventions which were successful in increasing the uptake of PA to patients with other conditions, such as those following orthopaedic surgery, were far less successful where those patients also had depression [30]. Lack of sufficient training for healthcare professionals in encouraging sedentary and depressed individuals to become physically active emerged as an important barrier [27, 33]. Even if such training was offered, heavy workload [32], the service’s performance targets [28], or qualification requirements [28], would take the priority. Staff turnover and absences presented additional barriers to the delivery of an intervention [32].

Overall, engagement of practitioners in delivering the interventions proved difficult [32]. This lack of engagement could also be attributed to some practitioners’ scepticism about the role of PA as an adjunct treatment for depression [32]. It could also be associated with the individuals’ preference for psychological treatments [32]. Furthermore, healthcare-grounded interventions faced additional challenges such as lack of appropriate infrastructure. Working with individuals from ethnic minorities who do not speak English with sufficient fluency was reported as a barrier to their engagement [26].

A number of interventions, which employed a motivational component, reported poor treatment fidelity [28, 29, 32].

Individual-related barriers included difficulties in accessing services [33], financial constraints [33], lack of time [29, 33], the nature of the condition [32], and cold and wet weather [24].

Design elements of the interventions, such as the lack of measurable goals were also identified as barriers [29]. In one study the computer interface used to deliver the intervention was perceived as insufficiently engaging [25]. In another study the intervention booklets were reported to be potentially overwhelming for the patients and perceived as physically too heavy to be carried by the practitioners [32].

Enablers to the uptake of PA amongst individuals with MMD included: the calming effects of PA [24]; participants’ satisfaction with interventions components such as the use of a diary to monitor adherence and progress [32]; the use of pedometers [25]; the presence of a gym instructor [31], and increased confidence in using gym equipment and in exercising safely [29].

Walking was found to be the preferred form of PA amongst some study participants e.g. [24]; other favoured activities included exercising in the gym and gardening [32]. In general, group-based PA was preferred [31]. For a full list of barriers and enablers identified in the studies see Appendix 1 (Table 4).

### Consultation

The results of the literature review were discussed with the stakeholder groups. Much focus was given to the barriers to PA that feature strongly for those with MMD. Here we found it useful to distinguish motivation from volition.

#### Motivation and volition

Gollwitzer makes a distinction between goal intention and implementation intention and explains that adopting behaviour has at least two distinct phases [34, 35]. Goal intention is the initial phase and is also termed motivational; during this phase the individual weighs up the costs and benefits of the proposed action. The second implementation phase is termed volitional; during this phase the individual develops the strategies and plans to implement the proposed action. Those suffering from depression demonstrate changes in executive brain functions [36], which impair their motivational and volitional capacities [37, 38].

Those with milder and moderate forms of depression are likely to suffer from volitional deficits [39]; they are likely to develop intentions, e.g. to engage in various activities, but are likely to show deficits in their planning abilities and execution [39]. Even where those with MMD are convinced that PA is worthwhile for them, they may not feel it is a possibility. This might be because they have an enhanced sense of the barriers, what might be called the “yes-but” problem, or it might be because their condition inhibits their ability to create a plan of action of the time required to start PA.

Because hopelessness escalates with severity of depression, those with more severe forms of depression are likely to show more motivational deficits; they are unlikely to develop new intentions [39–41]. Expectations that the behaviour will result in a desired outcome (outcome expectations), and the belief that one can perform the behaviour (self-efficacy), are therefore likely to be low amongst those suffering from depression, making them less likely to develop intentions to set and achieve health behaviour goals [38]. The findings from our consultation exercise revealed similar results.

#### Motivation

Motivation to act may be intrinsic, led by internally rewarding ends-in-themselves, or extrinsic, led by external rewards [42–45], or a means-to-an-end. Intrinsic motivation is associated with individuals’ tendencies to be interested in and engage with the world, and to develop their skills and knowledge even in the absence of external rewards [46].

Whether an action is seen as worthwhile is largely a product of the individual’s perception of risks and benefits, be they intrinsic or extrinsic. Stakeholders felt that MMD can distort this perception, making the risks greater, the benefits smaller. This led to the general point, repeated throughout the discussion, that interventions which work to increase PA in the general population were unlikely to work unless they included elements addressing MMD itself. For example, the person with MMD might acknowledge that PA is worthwhile for most people but not them, for example because they cannot imagine themselves as anything other than depressed.

Moving on to points that apply in relation to PA for all people, there was a discussion of the reasons that people might find PA worthwhile; in some cases it might be the sociability of the activity, the possibility of finding networks, whilst others might prefer lone activity. And clearly, there would be preferences in terms of types of activity. In terms of sociability, attractiveness and enjoying an external environment, walking emerged as a favoured type of PA. Unlike other treatments for MMD, PA is well-suited for offering intrinsic as well as extrinsic motivation, as the discussion in the group showed. This might lie behind its effectiveness as a treatment and can be used to advantage in developing PA as a treatment and in order to encourage its uptake. PA itself can become motivating. Here an interesting question was whether non-physical activity rather than PA, such as social meetings with peers, would have less success in improving MMD.

Affordability and accessibility were thought to be an issue for people in deprived communities such that interventions might improve the health of the financially better off more than the financially worse off; a problem sometimes termed intervention-generated inequality. Indeed, city-level approaches to increasing PA levels tend to be more effective amongst those who are already active or have showed an interest in being active. Encouraging the uptake of PA amongst sedentary individuals, particularly in deprived areas, is always a challenge to Public Health [47].

Participants suggested that MMD often came in cycles and that initiatives would be unlikely to succeed when people were at their lowest point in the cycle; as such, initial failures to encourage PA should not prevent further attempts. Again, this also suggested the importance of treating MMD using other treatment methods alongside the PA.

#### Volition

Participants felt that MMD could interfere with volition such that even if they were persuaded that taking up PA was worthwhile, their ability to execute the plan to do so could be impaired. Barriers here would be largely internal, emphasising again the need to treat MMD within the PA program. In addition, the promotion of small amounts of activity, such as three-minute walks, might be more effective; or the promotion of activity through indirect means, such as short but frequent health appointments.

## Discussion

This scoping review had five objectives, four of which have now been addressed. The fifth aim was to develop an initial conceptual model of how interventions might work in increasing an uptake of PA amongst those with MMD. Given the limited evidence found in the review, the model should be viewed as of the ‘how-possibly’ rather than ‘how-actually’ type [48]; in other words, a model of how the various interventions might work rather than how they are known to work.

### Stage 7: The development of a conceptual model

**Fig. 2:**
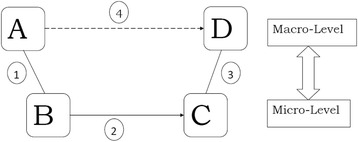
Coleman’s boat

The findings from this scoping study illustrate that both motivational and volitional deficits as well as social and environmental factors may impede an uptake of PA amongst those with depression. One way of modelling this uses Coleman’s model (or “Coleman’s Boat”) as a framework (please see Fig. 2 below); this explicitly takes account of the social context in social change (such as behaviour change), as well as paying attention to the specific nature of the individual [49–51].

Coleman’s purpose with the model was to show how social change occurs at micro and macro level, where micro level is usually taken to be that of the social individual or agent, and macro level, that of groups such as institutions. Hence, Node A represents a social fact at macro level; node B represents a fact at micro level concerning the “desires, goals, values, preferences, motives, emotions, habits […], routines, scripts, heuristics, cognitive schema, and identities […] of the individual agent” ([50] p.6). Nodes C and D represent the micro and macro levels (respectively) after the change has occurred. The lines between A and B (line 1) and C and D (line 3) represent the link between the macro and micro level; this is usually two way with, for example, the macro structures affecting how individuals at the micro level think and decide and vice versa. Line 2 represents the change at micro level, typically the behaviour of individuals; line 4 represents the change at macro level, for example, as one structure is either reinforced or modified. Change at the macro level is usually or always mediated via individual behaviour, hence line 4 is a dotted rather than unbroken line.

For our purposes, then, node A can be conceptualised as the population with MMD and their social situation including an intervention delivered by, for example, those diagnosed with the condition by their general practitioners. At node B, those with MMD are conceptualised as individuals having inter alia impaired ability to engage in PA over and above those individuals in the general population. The intervention that is delivered aims to reduce or overcome this deficit. If the intervention is successful, then changes occur at micro and macro level (lines 2 and 4) resulting in individuals motivated to do PA (node C) and, more widely, an increase in PA in the population with MMD (node D). In the light of the wider insights of the scoping review, the individual level (B) MMD would be conceptualised as individuals with motivational and volitional deficits to overcome, such as the weakened ability to plan action. Furthermore, the macro-micro level relationship between nodes A and B would include the effect they might have on each other; for example, an individual who becomes motivated to take up PA may live in a cultural environment (macro level fact), where some or even most of such activity is not seen as culturally acceptable. Hence, motivational-based interventions might be least successful in the communities where it is most needed, particularly economically deprived communities where levels of MMD are higher [52, 53].

At the micro level, one implication is that it would be worthwhile to combine interventions to increase an uptake of PA amongst those with depression with psychological treatments for MMD such that the approaches complement each other; an example might be a course of CBT, combined with a series of exercise classes or a walking group. One study that employed one of these approaches (CBT) was successful in both increasing uptake of PA and alleviating depression symptoms [26].

Volitionally, interventions may also be able to increase PA in those with MMD through indirect means; for example, frequent short therapy sessions (micro level) may be better than long infrequent sessions because they require more physical activity from the patient [54]. Furthermore, a 2013 Cochrane Review identified that more frequent sessions have a larger effect on mood [54].

Individuals’ intentions to be active can also be enhanced or impeded by the social context of interventions. The findings from our consultation exercises highlighted the importance of social relations in the formation of intention to be active. This finding is consistent with previous studies on PA attrition rates [55], indicating that, amongst other factors, social support from family and families’ attitudes towards PA had a significant effect on participation in PA. Amongst reviewed studies, some commentaries emphasised the importance of the social environment for PA [56]. Only one study, however, attempted to evaluate the effects of significant others’ support on individuals’ willingness to take up exercise on prescription. In our model, the social context’s effect on PA uptake represents a macro-level fact affecting a micro-level change (the individual’s ability to uptake PA).

The social context and its effect on volitional deficits may have been a factor in a study that was apparently focusing only on motivational deficit [31]. This was where a group instructor would help overcome volitional deficits, by instructing and supervising gym sessions. Other studies also report that the characteristics of group-based physical activity, such as bonding between group members, can evoke a sense of obligation and unwillingness to let others down by not showing for the PA [57].

Whilst supervised PA can overcome volitional issues there is a problem of dependence. In a study exploring adherence to PA post-supervised interventions for individuals with first-episode psychosis (FEP), adherence to unsupervised exercise was low [58]. It might be that supervised PA programmes lead to a certain level of dependency on exercise professionals for support. Also, low adherence to exercise post-intervention might result from interventions which fail to increase self-efficacy sufficient for physical activity maintenance (PAM). This highlights the importance of peer-group support or volunteers’ engagement in intervention to increase PA levels to ensure their long-term sustainability.

Nodes A and B on the model draw attention to the physical as well as the social environment. The findings from our consultation exercises and empirical evidence from behavioural economics highlight the importance of the environment in the choices we make (so-called nudge theory) [59]. For example, the results from our study confirm the importance of geographical proximity of sport facilities or parks for both the uptake and maintenance of PA [60, 61]. Although as Walking for Health [57] illustrates, the physical proximity of physical activity location may become less of an issue once the relationships between PA group members are established.

Our study participants indicated the importance of cost and convenience in facilitating an uptake of PA. This finding is similar to findings from previous studies, indicating that unaffordable facilities are the key barrier to PA amongst ethnic minority groups [62], and difficulties in engaging individuals in PA who live in deprived areas [63]. Issues such as unavailable childcare, personal safety and cultural inappropriateness of activities, were identified in previous studies as barriers to PA [62].

This model, then, would encourage the development of interventions which take in the motivational and volitional picture of action, combined with a complex view of the relationship between micro and macro environments. Individuals will vary widely, both in the balance of their own motivational and volitional attitudes to PA and in such matters as their socio-cultural environment. An individualised plan might work best [37] but if not possible, at least an awareness of the need to cover a variety of factors should help practitioners to develop more effective interventions.

Point D is the successful uptake of PA in a group of people with MMD exposed to the intervention at AB. It is not simply the addition of numbers of individuals at point C, those who have decided, or are inclined, to take up PA. This is because of feedback loops both here and at other points in the model. For example, the stability of the individuals’ intentional states regarding PA may be undermined by the macro environment through, say, physical or social barriers. Alternatively, the feedback may be positive as when the sociability of the activity is an important part of its appeal. Another positive feedback may rest in the type of PA; a combination of resistance and mixed training were found to be more effective than aerobic exercise [42]. This suggests the importance of selecting the most appropriate types of PA for those with MMD. Our study identifies walking and for some, gym-based activities and gardening as preferable forms of PA.

*Coleman’s model:* Coleman’s model can be, and has been here, used to represent a mechanism of change. Broadly, it shows how the intervention can be represented as micro and macro facts and/or factors that change the structure of individuals’ motivation and volition, as well as physical and social environments, causing behaviour change which can be maintained to the level of a social change. It is intended to be a simple picture, bringing out the chief shortfalls of current interventions. The model might be used in tandem with the health psychology model, Health Action Process Model (HAPA), as the latter includes constructs, such as self-efficacy, which will be of use in constructing and evaluating complex interventions [37, 38]. In this study we used exclusively the Coleman model since it is strong in enabling the picturing of the various ways or mechanisms by which an intervention can work or not. Furthermore, it allows us to take into account the social and environmental factors which may affect an uptake of physical activity.

*Strengths and limitations:* The validity of this study was achieved by: i) providing the details of the study process, including study selection, data extraction and data analysis; ii) ensuring that exclusion and inclusion criteria were applied independently by two researchers; and iii) involving the involvement of individuals with experience of depressionthroughout the study. One limitation of the review is that the quality of intervention design and evaluation was not formally assessed. This is appropriate for the objectives of a scoping review. Having established the extent and potential value of the included literature it would now be beneficial to further assess the quality of included studies within a formal systematic review process.

*Implications for policy and practice:* This study builds upon the developing body of knowledge in relation to an uptake of PA and MMD. Although the review could not settle the question of which approaches and interventions are effective in increasing an uptake of PA amongst individuals experiencing MMD, it has enabled the development of an explanatory model that can inform practice, policy and research. For practice and policy, this is mainly through highlighting the need to consider a broad range of mechanisms through which interventions work or fail to work in increasing an uptake of PA in those with MMD and whether different approaches may be effective for different subgroups of individuals with MMD.

*Research implications:* The model presented is of a how-possibly type, a framework of a hypothetical mechanism by which interventions would lead to changes in behaviour regarding uptake of PA. Researchers, particularly those of realist bent, would test this model looking at, for example, how the intervention at macro level is perceived at micro level, and whether the hoped-for changes in motivation and volition are actually seen. The need to look at volition is a clear implication of the model. In this way, the model would be developed from “as-if” to “as-actually”, that is, as shown by evidence.

This model could be developed for different contexts, such as environmental, social or ethnic groups. It was noticeable that the studies reviewed had little focus on socio-demographic factors and other sub-categories; these were collected inconsistently. Two studies listed sub-categories which were collected, without providing further details; seven studies reported the sub-categories to describe the baseline characteristics of participants. However, only two studies explored how the sub-categories could have affected the results [26, 30]. In one study those who continued to participate in the intervention were younger than those who dropped out [30]. In the other study those who provided follow-up data at 12 months had higher incomes [26]. [See Appendix 2 - Table 5 - Modifiers of Change]. As such, the proposed modified research would evaluate programs across a wider range of outcomes than whether they succeeded in increasing an uptake of PA, instead taking in such matters as how they worked, through what mechanisms, and for whom – this is, of course, a broadly realist approach, which would seem appropriate in this complex area. One suggestion from the consultation group of those with the condition is noteworthy for both future research and practice; this is the cyclical nature of the condition. This adds to the complexity, of course, but it also provides opportunity if this cycle is included in considering not just what type of interventions are effective and with whom, but also when. This is something which to our knowledge has not been noted in previous studies.

## Conclusions

Given the strength of evidence favouring PA as a treatment for MMD, the need for equally strong evidence for delivering this treatment is urgently needed by practitioners and commissioners. At present, there is a shortfall in evidence. This study suggests, however, that attendance to the volitional as well as motivational deficits in MMD would be worthwhile in any programmes to increase PA in that population. Similarly, the environmental and social contexts of interventions also need attention.

## Appendix 1

**Table 4:** Barriers and enablers to implementation of the intervention (the system and organisational level) and to the uptake of PA (individual-related levels)

1. Crone, D., Johnston, L.H., Gidlow, C., Henley, C., James, D.V.B. [33].
A) Barriers
*i) Organisational & system level*
• Physical activity referral scheme is less suited to the needs of MMD patients. • Those with mental health problems find that there are barriers impeding their ability to access health services [53]. • Primary health care professionals are insufficiently trained to work with patients affected by mental health issues.
*ii) Provider/practitioner level*
• Some healthcare professionals remain skeptical of the role of physical activity as an adjunct treatment for those with mental health problems [54].
*iii) Individual level*
• The uptake of the scheme was significantly lower in the mental health referrals. • Difficulties in access. • Financial constraints. • The side effect(s) of antidepressants [53]. • Lack of social network and support.
B) Enablers None listed.
2. Duda, J., Williams, G., Ntoumanis, N., Daley, A., Eves, F., Mutrie, N., Rouse, P.C., Lodhia, R., Blamey, R.V., Jolly, K. [28].
A) Barriers
*i) Organisational & system level*
• Insufficient training for practitioners delivering the intervention. • A low training attendance due to work-related commitments. • Lack of infrastructure e.g. limited access to PCs making it difficult for practitioners to watch training videos or receive email reminders sent by the research team.
*ii) Provider/practitioner level*
• Poor treatment fidelity (e.g. an inadequate provision of autonomy support).
*iii) Individual level*
• Poor engagement with minority ethnic communities, who do not speak English with sufficient fluency.
*iv) Intervention level*
• Practicalities of organising an intervention: the use of interpreters proved challenging.
B) Enablers: None listed
3 Forsyth A., Deane F.P., Williams P. [24].
A) Barriers
*i) Organisational & system level*
• Engaging healthcare staff in the delivery of the intervention.
*ii) Provider/practitioner level* None listed.
*iii) Individual level*
• Engaging patients proved difficult and approximately 50% of all appointments were either cancelled or missed.
*iv) Intervention level* None listed.
B) Enablers
*i) Intervention level*
• Calming effects of PA. • A preferred form of PA was walking.
4 Littlecott, H.J., Moore G.F., Moore, L., Murphy S. [29].
A) Barriers
*i) Organisational & system level None listed.*
*ii) Provider/practitioner level*
• Poor treatment fidelity e.g. motivational interviewing and goal setting were not fully delivered [58].
*iii) Individual level*
• Time and costs. • The nature of the intervention.
*iv) Intervention level*
• Intervention design: lack of measurable goals might have led to reduced self-efficacy.
*v) Context level*
B) Enablers:
*i) Intervention level*
• Increased participants’ confidence in using gym equipment and in exercising safely.
*ii) Social context*
• Family can positively impact the participants’ engagement with the interventions; friends do not seem to have a similar impact.
5 Mailey E.L., Wójcicki T.R., Motl R.W., Hu L, Strauser D.R., Collins K.D., McAuley E. [25].
A) Barriers
*i) Organisational & system level None listed.*
*ii) Provider/practitioner level None listed.*
*iii) Individual level*
• Poor engagement with PApost intervention. • Participants’ self-efficacy declining over time.
*iv) Intervention level*
• Inadequate intervention interface design.
B) Enablers
*i) Intervention level*
• Participants’ satisfaction with a number of intervention components such as meetings with intervention staff or using pedometers.
6 Oeland A.M., Laessoe U., Olesen A.V., Munk-Jørgensen P. [31].
A) Barriers
*i) Organisational & system level None listed.*
*ii) Provider/practitioner level None listed.*
*iii) Individual level*
• A low uptake of amongst patients suffering from ill mental health. • In the follow-up period improvement stops.
*iv) Intervention level*
• Post-intervention, levels of PA decrease over time e.g. due to the lack of professional instructions.
B) Enablers:
*i) Intervention level*
• The presence of the instructor. • Mode of deliver: group exercises (which might have heightened motivation as a result of social interactions).
7 Pentecost C., Farrand P., Greaves C.J., Taylor R.S., Warren F.S., Hillsdon M., Green C., Welsman J. R., Rayson K., Evans P.H., Taylor A.H. [32].
A) Barriers
*i) Organisational & system level*
• Staff turnover and absences. • Heavy workload. • Clinical work and the service’s performance targets take the priority over intervention delivery
*ii) Provider/practitioner level*
• Practitioners and participants’ preference for psychological treatments. • Practitioners not giving information booklets. • Poor treatment fidelity; deviations from the intervention delivery protocol.
*iii) Individual level*
• The nature of the condition resulting in unwillingness to engage in PA.
*iv) Intervention level*
• Intervention design; information booklets –potentially overwhelming. • Information booklet being too heavy:*‘I must admit, because I have so much to carry as a PWP, it was a bit too much’.*
*v) Extraneous circumstances*
• Illness of a member of the research team.
B) Enablers
*i) Intervention level*
• Behavioural Activation and PA enhancing recovery rates. • Information booklets.. • The diaries seemed to be one of the most useful tools in the booklets (to plan and monitor PAs). • Preferred types of PA: walking, gardening and exercising in a gym. • Monitoring PA levels with pedometers.
*i) Individual level*
• PA promotion was acceptable to patients.
8 Piette J.D., Richardson C., Himle J., Duffy S., Torres T., Vogel M., Barber K., Valenstein M. [26].
A) Barriers
*i) Organisational & system level: None listed.* *ii) Provider/practitioner level: None listed.* *iii) Individual level*
• an initial uptake: 32% of contacted individuals refused participation.
*iv) Intervention level*
• Underrepresentation of individuals from various ethnic minorities (16% of the • study population).
B) Enablers:
*i) Intervention level*
• The use of CBT to increase an uptake of PA*.*
9 Pomp S., Fleig L., Schwarzer R., Lippke S. [27].
A) Barriers
*i) Organisational & system level:* None listed. *ii) Provider/practitioner level:* None listed. *iii) Individual level*
• Individuals with depressive symptoms did not increase their exercise levels. • Individuals’ depleted self-regulatory resources resulting in fewer capabilities to implement health behaviour; they planned less; struggled to set realistic plans and to monitor their PA levels. Also, they did not adhere to their plans.
*iv) Intervention level:* None listed.
B) Enablers:
*i) Intervention level*
• Participants suffering from depression may benefit from weekly reminders and booster sessions; also, from additional psychotherapeutic support such as Cognitive behavioural therapy (CBT). • Integrated approaches that address the management of depressive symptoms and health behaviour.
10 Suija K., Pechter U., Kalda R., Tähepõld H., Maaroos J., Maaroos H.I. [18]
A) Barriers
*i) Organisational & system level:* None listed. *ii) Provider/practitioner level:* None listed. *iii) Individual level*
• Lack of time. • The rainy and cold weather.
*iv) Intervention level*
• Type of PA intervention: unsupervised home-based exercise*.*
B) Enablers:
*i) Individual level*
• Positive PA experience.

## Appendix 2

**Table 5:** Modifiers of change

Study	Demographic data & other potential moderators of change	How the sub-categories have been used in the studies
Forsyth et al., 2009 [64]	Gender, Age, BMI	The authors report which sub-categories have been collected, without providing any further details.
Mailey et al., 2010 [25]	Gender, Age, Ethnic group	The data have been used to describe the sample of participants.
Oeland et al., 2010 [31]	Gender, Age, BMI, VO_2_ max	The authors report which subcategories have been collected. They excluded from the study those who had a BMI > 35.
Pentecost et al., 2015 [32]	Gender, Age BMI, Ethnic group, relationship status, smoking status, postcode, number of dependents and age upon leaving full-time education	Usable descriptive data were reported for 28 (47%) participants. Only 11 participants (37%) at baseline and 9 (30%) at the 4-month follow up provided data for BMI and BP.
Piette et al., 2011 [26]	Gender, Age, Ethnic Group, relationship status, Education, Employment Status; annual household income, BMI, Diabetes Medication, Antidepressant medication	16% of participants were ethnic minorities, however, no other information about this could have affected the uptake of PA was provided. The authors identified that there were differences between those who provided follow-up data at the 12-month follow up; they had higher income.
Suija et al., 2009 [27]	Gender, Age, BMI, Physical Activity level, Smoking status, antidepressant medication	The baseline characteristics of participants have been reported. The authors haven’t discussed, however, how these sub-categories could have affected the uptake of PA.
Crone et al., 2008 [33]	Gender, Age	This was the UK’s PARS study. Women made up the majority (64%) of patients referred to scheme due to mental health. The average age of “mental health participants” was significantly lower than “physical health participants” (42 ± 14 year versus 51 ± 14 years; *p* < 0.0001). Fewer patients with mental health problems (60%) took up referral at the local leisure centre, compared to those with poor physical health (69%). The authors refer to their previous studies related to the provision of PA for patients with mental health problems; where one financial constraint (sub-category – household income), was a reason for mental health patients dropping out from PARS. p. 1093.
Duda et al., 2014 [28]	Gender, Age, Ethnic Group, Qualifications, alcohol intake	The sub-categories are reported in the article, but how they might have affected the uptake of PA isn’t. The authors state: “The city in which the trial took place has a relatively young, ethnically diverse population, with about third of the people non-white [32] and 16.5% born outside the UK at the 2001 census.” P. 4 They also add report that the recruitment to the study was challenging due to the ethnic diversity of the sample, resulting in difficulties in administering the study questionnaire to people who do not speak English.
Littlecott et al., 2014 [29]	Gender, Age, Level of Deprivation, Baseline Activity level	The baseline characteristics of participants have been reported. The authors haven’t discussed, however, how these sub-categories could have affected the uptake of PA. This was the UK’s study that identified effects of PA for patients with CHD risk only, mediation analyses were limited to this subsample.
Pomp et al., 2013 [30]	Gender, Age, Marital Status, Educational Background, occupational status	The differences between groups (participants in the intervention and control arms) at T1 were found of physical activity and educational background. The participants did not differ with regard to sex, age, and occupational status. The results revealed that those who continued to participate in the study were younger than those who dropped out. They did not differ, however, in terms of gender, occupational status, high school degree, partner status (between T1 and T3).
